# Examining the role of intrinsic and reflexive contributions to ankle joint hyper-resistance treated with botulinum toxin-A

**DOI:** 10.1186/s12984-023-01141-8

**Published:** 2023-02-07

**Authors:** Ronald C. van’t Veld, Eline Flux, Wieneke van Oorschot, Alfred C. Schouten, Marjolein M. van der Krogt, Herman van der Kooij, Marije Vos-van der Hulst, Noël L. W. Keijsers, Edwin H. F. van Asseldonk

**Affiliations:** 1 Department of Biomechanical Engineering, University of Twente, Enschede, The Netherlands; 2 Department of Rehabilitation Medicine, Amsterdam Movement Sciences, Amsterdam UMC, Vrije Universiteit Amsterdam, Amsterdam, The Netherlands; 3 Department of Research, Sint Maartenskliniek, Nijmegen, The Netherlands; 4 Department of Biomechanical Engineering, Delft University of Technology, Delft, The Netherlands; 5 Department of Rehabilitation, Sint Maartenskliniek, Nijmegen, The Netherlands; 6 Department of Rehabilitation, Cognition and Behavior, Donders Institute for Brain, Radboud University Medical Center, Nijmegen, The Netherlands

**Keywords:** Spasticity, BoNT-A injections, Joint resistance, Instrumented SPAT, System identification

## Abstract

**Background:**

Spasticity, i.e. stretch hyperreflexia, increases joint resistance similar to symptoms like hypertonia and contractures. Botulinum neurotoxin-A (BoNT-A) injections are a widely used intervention to reduce spasticity. BoNT-A effects on spasticity are poorly understood, because clinical measures, e.g. modified Ashworth scale (MAS), cannot differentiate between the symptoms affecting joint resistance. This paper distinguishes the contributions of the reflexive and intrinsic pathways to ankle joint hyper-resistance for participants treated with BoNT-A injections. We hypothesized that the overall joint resistance and reflexive contribution decrease 6 weeks after injection, while returning close to baseline after 12 weeks.

**Methods:**

Nine participants with spasticity after spinal cord injury or after stroke were evaluated across three sessions: 0, 6 and 12 weeks after BoNT-A injection in the calf muscles. Evaluation included clinical measures (MAS, Tardieu Scale) and motorized instrumented assessment using the instrumented spasticity test (SPAT) and parallel-cascade (PC) system identification. Assessments included measures for: (1) overall resistance from MAS and fast velocity SPAT; (2) reflexive resistance contribution from Tardieu Scale, difference between fast and slow velocity SPAT and PC reflexive gain; and (3) intrinsic resistance contribution from slow velocity SPAT and PC intrinsic stiffness/damping.

**Results:**

Individually, the hypothesized BoNT-A effect, the combination of a reduced resistance (week 6) and return towards baseline (week 12), was observed in the MAS (5 participants), fast velocity SPAT (2 participants), Tardieu Scale (2 participants), SPAT (1 participant) and reflexive gain (4 participants). On group-level, the hypothesis was only confirmed for the MAS, which showed a significant resistance reduction at week 6. All instrumented measures were strongly correlated when quantifying the same resistance contribution.

**Conclusion:**

At group-level, the expected joint resistance reduction due to BoNT-A injections was only observed in the MAS (overall resistance). This observed reduction could not be attributed to an unambiguous group-level reduction of the reflexive resistance contribution, as no instrumented measure confirmed the hypothesis. Validity of the instrumented measures was supported through a strong association between different assessment methods. Therefore, further quantification of the individual contributions to joint resistance changes using instrumented measures across a large sample size are essential to understand the heterogeneous response to BoNT-A injections.

**Supplementary Information:**

The online version contains supplementary material available at 10.1186/s12984-023-01141-8.

## Background

Botulinum neurotoxin-A (BoNT-A) injections are currently the most frequently used clinical intervention for focal spasticity [1–3]. Spasticity is a common symptom after various brain and neural injuries, such as spinal cord injury (SCI) or stroke, referring to an exaggerated stretch reflex, i.e. stretch hyperreflexia [4, 5]. Spasticity is perceived as an increased joint resistance to movement, i.e. joint hyper-resistance. BoNT-A injections are used clinically to reduce muscle activity and hence spasticity [1]. BoNT-A injections reduce muscle activity by inhibiting the release of acetylcholine at the neuromuscular junction, which chemically denervates the exposed muscle fibers. BoNT-A effects reduce after 2 to 4 months due to nerve sprouting and muscle re-innervation [1].

Clinical evaluation of BoNT-A injections has shown a significant reduction in joint resistance after 2–8 weeks using the modified Ashworth scale (MAS) [6–8]. With the MAS, currently a common clinical test, clinicians evaluate overall joint resistance, which can physiologically include tissue characteristics, and tonic and reflexive muscle activity [5, 9–11]. For the MAS, a single passive movement profile is repeatedly applied, whereas movements with varying characteristics, e.g. slow and fast velocities, are required to unravel joint resistance contributions. Therefore, the MAS can clinically only evaluate spasticity indirectly and cannot distinguish between spasticity and other symptoms as involuntary background activity, shortened soft tissue, contractures and muscle fibrosis [4, 12, 13]. Furthermore, the MAS has a questionable reliability, especially when applied at the lower limb [11, 14]. Hence, the clinical effect of BoNT-A injections on spasticity is poorly understood, while BoNT-A injections are a frequently used clinical intervention for spasticity.

Quantification of the intrinsic and reflexive contributions to joint hyper-resistance is essential to understand the beneficial and adverse effects of BoNT-A injections and support clinical decision making. BoNT-A injections can, for example, have side-effects and should ideally only be administered to patients who suffer from increased reflexive contributions to joint hyper-resistance [15]. Objective information on both intrinsic and reflexive joint resistance can support clinical decision making and help evaluate treatment effects [5]. The intrinsic resistance represents the combination of tissue-related non-neural and tonic neural contributions to joint resistance [10]. The reflexive resistance, representing the phasic neural contributions, can be used as measure for spasticity. Model-based processing of neuromechanical responses can be used to unravel and quantify the intrinsic and reflexive contributions [10, 16–20]. Furthermore, instrumentation and motorization using robotic devices can improve precision, consistency and objectivity of the applied movements and measurements [21–23].

Model-based evaluation of BoNT-A effects on joint hyper-resistance contributions have been applied using neuromechanical models [24–27]. These studies showed conflicting results on BoNT-A effects with either no change or a significant reduction of the reflexive resistance observed after injection. The neuromechanical modelling approaches used limited experimental datasets measured over the full passive range of motion (pROM), similar to current clinical measures. The subsequent joint resistance estimation primarily relies on a priori knowledge and simplifying assumptions. As a result, these methodologies are sensitive to incomplete model definitions and imperfect a priori knowledge [17, 18, 20]. Furthermore, the lack of a gold standard complicates interpretation of the reported conflicting results [5, 28, 29]. Besides the selected model, differences in reported BoNT-A effects may also be influenced by participant heterogeneity, the experimental setup, and the assessed joint. Given the conflicting results and lack of a gold standard, investigating fundamentally different approaches to assess joint hyper-resistance is of interest to improve understanding of BoNT-A effects.

An alternative approach to assess BoNT-A effects on joint hyper-resistance contributions is data-driven modelling. Data-driven modelling evaluation of BoNT-A effects on joint hyper-resistance contributions could be executed using system identification [10, 16, 30, 31]. For example, the parallel-cascade (PC) system identification technique has shown the ability to discriminate spastic participants from controls and paretic from non-paretic joints [30, 32]. The PC technique has also shown good group-level responsiveness during the evaluation of several clinical treatments, like functional electrical stimulation-assisted walking, Tizanidine and robot-assisted gait training [33–35]. Currently, no system identification results have been reported on BoNT-A effects. Contrary to neuromechanical modelling, the system identification techniques previously tested in a clinical setting used rich experimental datasets measured over only a limited portion of the pROM [30–35]. As intrinsic and reflexive joint resistance depend on joint angle, the obtained joint resistance estimates do not characterize the full pROM [36].

The goal of this paper was to distinguish the contribution of intrinsic and reflexive ankle joint resistance for participants treated with BoNT-A injections to reduce spasticity. We hypothesized that reflexive joint resistance decreases 6 weeks after injection, while returning close to baseline after 12 weeks [24, 25]. Due to the reduced reflexive joint resistance, we also expected the overall joint resistance to decrease 6 weeks after injection, while returning close to baseline after 12 weeks [6–8]. In absence of a gold standard, the joint resistance contributions were assessed using multiple joint resistance measures with different characteristics and limitations. Joint resistance contributions were estimated using clinical measures (MAS/Tardieu Scale) [9, 37], an instrumented spasticity test (SPAT) [22, 23] and a parallel-cascade (PC) system identification technique [10, 30]. To support validity of the measures used, the linear association between the various outcome measures was investigated.

## Methods

### Participants and study schedule

Six people with SCI and three stroke survivors participated in the study: age 54.4 ± 11.1 year, 2 women, see Table 1.

**Table 1:** Participant demographic, clinical and BoNT-A injection characteristics (*N = 9*)

Age	Gender	Diagnosis	Meas. side	Months post stroke/SCI	AIS (SCI)	BoNT-A injection	BoNT-A brand	BoNT-A dose per muscle (units)
54	M	Stroke (Ischaemic)	R	12		4th	Dysport	GM (300); GL (300)
58	M	Stroke (Ischaemic)	L	69		5th	Allergan	SOL (50); GM (50) GL (50)
49	M	Stroke (Hemorrhagic)	L	64		1st	Dysport	SOL (400); GM (200) GL (200)
67	M	SCI (C5–C7)	L	30	D	8th	Dysport	SOL (300)
62	F	SCI (T7–T12)	L	54	B	13th	Dysport	SOL (400); GM (200) GL (200); TP (200)
29	M	SCI (T7–T12)	R	25	A	4th	Dysport	SOL (200); GM (200) GL (100)
51	M	SCI (T7–T12)	R	183	C	3rd	Dysport	SOL (300); GM (200) GL (200)
59	M	SCI (L1)	L	144	C	7th	Dysport	SOL (150); GM (160) GL (160)
61	F	Cauda equina syndrome (L4–L5)	R	17		1st	Dysport	SOL (300); GM (200) GL (200); TP (300)

The (most) affected side with a pROM $\ge 20^{\circ }$ was selected as measured side during experiments

AIS: American Spinal Injury Association (ASIA) Impairment Scale; BoNT-A: Botulinum Neurotoxin type-A; GM: Gastrocnemius Medialis; GL: Gastrocnemius Lateralis; SCI: Spinal Cord Injury; SOL: Soleus; TP: Tibialis Posterior

Patients treated at the Sint Maartenskliniek, Nijmegen were assessed for eligibility by their rehabilitation physician. Inclusion criteria were: (1) adult, older than 18  year; (2) stable neurological condition in chronic phase, minimum 6 months post-lesion/-stroke; (3) a MAS or Tardieu score $\ge$ 1 for any of the m. triceps surae; (4) treatment of any of the m. triceps surae with BoNT-A injections aimed at spasticity reduction; and (5) pROM of the affected ankle joint in the sagittal plane $\ge$ 20$^{\circ }$. Participants were excluded if BoNT-A injections were combined with other treatments aimed at reducing spasticity. Note, included participants did typically receive the BoNT-A injections in combination with home stretching exercises in line with usual care. Participants gave written informed consent before definitive inclusion.

**Fig. 1:**
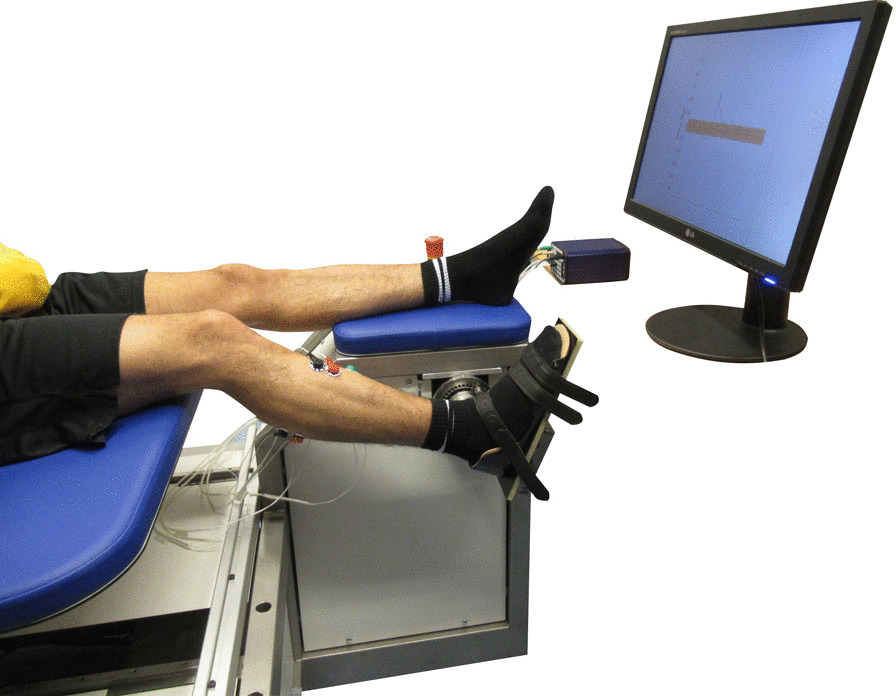
Experimental Setup. Participants were seated on an adjustable chair for the instrumented evaluations. The manipulator connected to the adjustable chair applied dorsiflexion, ramp-and-hold perturbations around the ankle joint, while measuring the biomechanical response. If the left foot was measured, the right leg was supported with a right lower leg support inserted into the chair frame (not shown)

In this exploratory longitudinal study, ankle joint resistance was evaluated across three sessions: a baseline (week 0) measurement on the same day as BoNT-A injection and two post-intervention measurements at 6 and 12 weeks after BoNT-A injection. The week 12 evaluation was usually measured on the same day as a new BoNT-A injection, as BoNT-A injections were repeated every three months. In each session the clinical evaluation was executed by the same trained physiotherapist (WO, non-blinded), whereas the instrumented evaluation was executed by a researcher (RV, EF or EA) using a robotic manipulator, see Fig. 1.

### Instrumented experimental setup

The instrumented evaluations (SPAT and PC technique) were performed with participants seated on an adjustable chair, see Fig. 1. The (most) affected side in compliance with the inclusion/exclusion criteria was measured. The measured foot was placed on a rigid footplate and secured using Velcro straps. The rigid footplate was part of the robotic manipulator fixed onto the frame of the adjustable chair. The chair supported the participant’s back and upper leg to achieve a fixed posture with 70$^{\circ }$ hip and 30$^{\circ }$ knee flexion. The hip and knee angles were selected to be attainable by all participants and to avoid muscle slack in order to allow for proper elicitation of the stretch reflex even with the small amplitude (2${^\circ }$) perturbations used for the PC technique [30, 36]. For each participant, the chair was adjusted to these hip and knee angles in the first session. For subsequent sessions, the chair was re-adjusted to the position of the first session to ensure constant posture across sessions. As such, the upper leg was firmly supported across all sessions to minimize movement of the leg that could introduce bias and variability in the instrumented measures. The ankle and manipulator axes of rotation were visually aligned by minimizing knee translation in the sagittal plane while rotating the footplate.

The robotic manipulator used a one degree-of-freedom actuator (MOOG, Nieuw-Vennep, the Netherlands) to apply the desired joint perturbations in the sagittal plane. Ankle angle and angular velocity were measured using an encoder situated at the actuator axis. Ankle torque was measured using a torque sensor placed between the actuator and footplate. The ankle angle, velocity and torque were recorded at 2048 Hz with the dorsiflexion direction defined as positive. For ankle angle, the neutral (0$^{\circ }$) angle was determined using a goniometer at 0$^{\circ }$ dorsiflexion/plantarflexion. For safety, manipulator movement was restricted to the maximal ankle pROM, which was re-evaluated every session, using adjustable hardware endstops. Measurements over full pROM (SPAT) were executed with a 2$^{\circ }$ margin at both endstops. Measurements over a limited pROM (PC technique) started 10$^{\circ }$ below the dorsiflexion endstop to avoid slack of the calf muscles. As pROM was re-evaluated every session, anatomical angles for both instrumented measurements could vary across sessions. At the start of each measurement, mean torque was measured over a 1 s period to determine the neutral (0 Nm) torque for that measurement.

### Experimental protocol

The same protocol was executed in all three sessions. A clinical evaluation was executed with participants lying supine on an examination table to obtain scores for the MAS (overall joint resistance) [9] and Tardieu Scale (reflexive joint resistance) [37]. During clinical evaluation, the knee was supported by a cushion to achieve 30$^{\circ }$ knee flexion, similar to the instrumented setup. For the MAS, the ankle joint was rotated three times over the full pROM in 1 s [9]. The MAS was scored on an ordinal six-point scale from 0, no increase in muscle tone, to 4, affected part(s) rigid in flexion or extension. For the Tardieu Scale, the ankle joint was rotated over the full pROM at three different velocities: V1, as slow as possible; V2, velocity approximately equal to limb falling under gravity; and V3, as fast as possible [37]. The quality ($TS_Q$) of the joint response was scored for all velocities on an ordinal five-point scale from 0, no resistance throughout the movement, to 4, infatigable clonus at a precise angle [37].

**Fig. 2:**
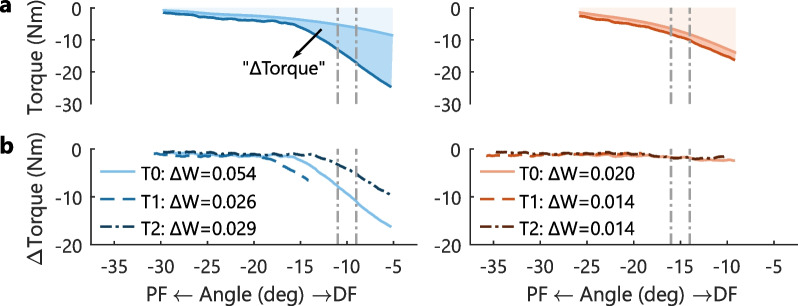
Instrumented SPAT assessment for two representative participants with a clear (left) and little (right) reflexive response. **A** Ensemble averaged (3 repetitions) torque-angle curves for the instrumented SPAT at both slow *(light solid line)* and fast *(dark solid line)* velocity at week 0 (T0). The work delivered by the ankle joint is highlighted for the slow velocity trial *(light-shaded area)* and the difference ($\Delta$Torque) between the fast and slow velocity trials *(dark-shaded area)*. The instrumented SPAT was analyzed from 10 to 90% pROM with the limited pROM used for the PC technique demarcated *(dash-dotted verticals)*. **B** Ensemble averaged difference in torque ($\Delta$Torque) between the fast and slow velocity SPAT at each session: week 0 (T0), 6 (T1) and 12 (T2) after BoNT-A injection. Torque differences were computed by interpolating the slow velocity torque data onto the exact angles measured in the fast velocity dataset, visualized by the dark-shaded area in **A**. Manipulator movement was restricted to the maximal ankle pROM each session for safety, reflected by the varying pROM depicted across sessions

The instrumented SPAT evaluation consisted of two measurements at different velocities emulating V1 and V3 of the Tardieu Scale, see Fig. 2A [22, 23]. First, three slow (10$^{\circ }$/s) dorsiflexion perturbations over the full pROM were applied. Second, three fast (150$^{\circ }$/s) dorsiflexion perturbations over the full pROM were applied. At both velocities, repetitions were separated by 20s of rest. The maximum dorsiflexion angle was held for 1s before returning towards plantarflexion with an opposite profile to the dorsiflexion perturbation. Participants were instructed to relax and not respond to the perturbations.

**Fig. 3:**
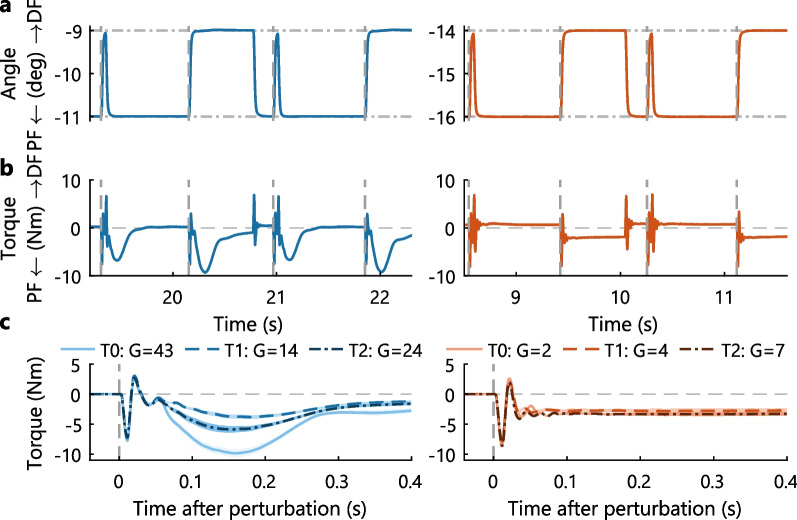
PC technique assessment for two representative participants with a clear (left) and little (right) reflexive response. **A** Four consecutive dorsiflexion perturbations *(onset at dashed verticals)* used for the PC technique at week 0 (T0). Perturbation signals were randomly generated, hence the different time-axes used to visualize a similar sequence of pulse and step perturbations. **B** The subsequent ankle joint response, measured as torque, elicited through each dorsiflexion perturbation. **C** Ensemble averaged (± SD, single measurement block) torque response at each session. The torque ensemble averages were created by aligning all step perturbations at the perturbation onset *(dashed verticals)*. The reflexive gain *G* (Nm s/rad) shows the quantified reflexive contribution at each session. To enhance visualization, torque ensembles were normalized to zero torque at perturbation onset

The PC technique evaluation consisted of two measurement blocks (2min) with 1min rest in between. In each block, a series of small (2$^{\circ }$ amplitude) ramp-hold-return perturbations were continuously applied, see Fig. 3A [38]. These ramp-and-hold perturbations had a 125$^{\circ }$/s max. velocity, 15800$^{\circ }$/s$^2$ max. acceleration and 40ms duration. Perturbations randomly switched between ’steps’, i.e. the maximum dorsiflexion angle was held for 580 ms, and ’pulses’, i.e. no hold period at the maximum dorsiflexion angle [39]. The manipulator returned towards plantarflexion with an opposite profile to the dorsiflexion perturbation. Participants were again instructed to relax and not respond to the perturbations.

### Data analysis

All data was analyzed using Matlab 2017b (Mathworks, Natick, MA, USA). For the instrumented SPAT, the work, i.e. product of force and displacement, around the ankle was used to quantify joint resistance [22, 23]. Work was computed as area under the torque-angle curve, ranging from 10% to 90% pROM. The torque-angle curve was corrected for gravitational effects of the footplate and foot. Work was computed as measure of: (1) intrinsic joint resistance from the slow velocity trials $W_{slow}$; (2) overall joint resistance from the fast velocity trials $W_{fast}$; and (3) reflexive joint resistance from the difference between the fast and slow trials ${\Delta }W$. All values of work were normalized for body weight (kg) and pROM. Due to a calibration issue, instrumented SPAT outcomes for the session at week 12 of one participant were removed.

For the PC technique, intrinsic and reflexive joint resistance parameters were estimated using a time-invariant algorithm modified from the original algorithm by Kearney et al. [10]. The algorithm consisted of the following steps:

1. The measured angle, velocity and torque signals were anti-alias filtered (2nd-order, 65.8 Hz, critically-damped) and downsampled to 146.3 Hz.
2. Measured acceleration was extracted from the state vector of the velocity low-pass filter and also downsampled to 146.3 Hz.
3. Non-parametric estimation of intrinsic, reflexive and voluntary torque contributions were obtained via an iterative procedure. Iterations continued until variance accounted for (%VAF) did not improve (< 0.005%) or reached max. 10 iterations.
  - Residual intrinsic torque was computed by subtracting reflexive and voluntary torque from the net torque. (${{1}^{st}-iteration}$) Reflexive and voluntary torque were set to zero.
  - A 35 ms intrinsic impulse response function (IRF) was estimated using a correlation-based method between angle and residual intrinsic torque. A pseudo-inverse approach based on minimum description length was used to retain only significant terms [40].
  - Residual reflexive torque was computed by subtracting voluntary and intrinsic torque, i.e. the convolved intrinsic IRF with angle, from the net torque.
  - A 650 ms reflexive IRF was estimated between half-wave rectified velocity and residual reflexive torque using the same correlation-based method.
  - Residual voluntary torque was computed by subtracting intrinsic and reflexive torque, i.e. the convolved reflexive IRF with half-wave rectified velocity, from net torque.
  - Voluntary torque was estimated as the low-pass filtered (2nd-order, 0.5 Hz, Butterworth) residual voluntary torque.
4. The intrinsic inertia *I* (acceleration-component), damping *B* (velocity-component) and stiffness *K* (angle-component) were estimated using linear least squares between acceleration, velocity and angle, and intrinsic torque.
5. The reflexive IRF was fit between half-wave rectified velocity and reflexive torque with both signals low-pass filtered (2nd-order, 14.6 Hz, critically-damped).
6. The reflexive delay $\delta$ was estimated via a grid search (35–65 ms, 1 ms increments), coupled to a nonlinear least squares fit on the reflexive IRF of reflexive gain *G*, damping $\zeta$ and frequency $\omega$.

### Statistical analysis

The statistical analysis was performed using Matlab 2017b and R3.6.2 (R Foundation for Statistical Computing, Vienna, Austria). The outcome measures included two clinical measures (MAS, $TS_Q$), three instrumented SPAT measures ($W_{fast}$, ${\Delta }W$, $W_{slow}$) and three PC technique measures (*G*, *K*, *B*). $TS_Q$ was evaluated based on the highest velocity (V3) assessment of the Tardieu Scale only, as this velocity was closest to the instrumented evaluations.

On an individual level, the hypothesized longitudinal BoNT-A effects were evaluated by comparing the measured resistance between baseline and week 6, as well as between week 6 and week 12. For each outcome measure, we considered the hypothesized BoNT-A effect observed, if a reduced resistance compared to baseline was measured at week 6 in combination with a return towards baseline at week 12. On group-level, the hypotheses on the longitudinal BoNT-A effects were evaluated using the Friedman non-parametric one-way repeated measures analysis for all outcome measures [27]. Post-hoc multiple comparison tests between sessions were executed for significant Friedman test results. For each multiple comparison, *p*-values were adjusted using the Bonferonni correction. Significance level was set at $\alpha$ = 0.05.

To support reliability of the longitudinal BoNT-A evaluation, repeatability of the instrumented measures was assessed using the intraclass correlation coefficient (ICC) [41]. ICCs were computed with a two-way mixed effects model, assessing absolute agreement between single repetitions. ICC robustness was investigated using the 95% confidence interval (CI) constructed via a non-parametric bootstrap procedure using the bias corrected and accelerated (BCa) method [42]. Minimal Detectable Difference (MDD) was calculated using the IC according to [43].

Validity of the outcome measures was assessed based on linear associations. We expected strong ($r>$ 0.7) linear associations between outcome measures estimating the same contribution, i.e. between the overall measures (MAS, $W_{fast}$), the reflexive measures ($TS_Q$, ${\Delta }W$, *G*) and the intrinsic measures ($W_{slow}$, *K*, *B*). Furthermore, we expected no or weak linear associations between outcome measures estimating different contributions. The non-parametric Spearman’s rank correlation coefficient $\rho$ was used for associations involving the ordinal clinical measures. Pearson’s correlation coefficient *r* was used for associations involving only instrumented measures. Robustness of $\rho$ and *r* were investigated using the 95% CI based on a BCa bootstrap procedure.

## Results

We investigated BoNT-A effects on the intrinsic and reflexive contributions to ankle joint hyper-resistance in nine participants at three sessions: week 0 (T0), 6 (T1) and 12 (T2) after BoNT-A injection. Joint resistance was assessed using common clinical measures, i.e. MAS, Tardieu Scale ($TS_Q$), an instrumented SPAT ($W_{fast}$, ${\Delta }W$, $W_{slow}$) and PC system identification technique (*G*, *K*, *B*).

### Qualitative analysis of instrumented measures

The reflexive response elicited during the instrumented evaluation strongly varied between participants. For example, some participants showed a clear reflexive response in both instrumented measures, whereas other participants showed a small or no reflexive response, see Figs. 2A, 3B. This heterogeneity in the reflexive response was observed both before and after BoNT-A injection, see Figs. 2B, 3C. For the instrumented SPAT, the reflexive response was mainly present in the part of the pROM close to maximum dorsiflexion, see dark-shaded area Fig. 2A. For the PC technique, the reflexive response was observed 100–300ms after each dorsiflexion perturbation, see Fig. 3B, C.

The observed intrinsic response also varied between participants. For the instrumented SPAT, variation of the intrinsic response was seen over the full pROM, see light-shaded area Fig. 2A. For the PC technique, variation of the intrinsic response was visible in the sustained plantarflexion torque response after step perturbations (i.e. a 580 ms hold period at maximum dorsiflexion), see Fig. 3B. This spring-like behavior around the joint, especially visible in the absence of a reflexive response, was interpreted as the elastic intrinsic resistance, i.e. intrinsic stiffness.

### Longitudinal evaluation of BoNT-A injections

**Fig. 4:**
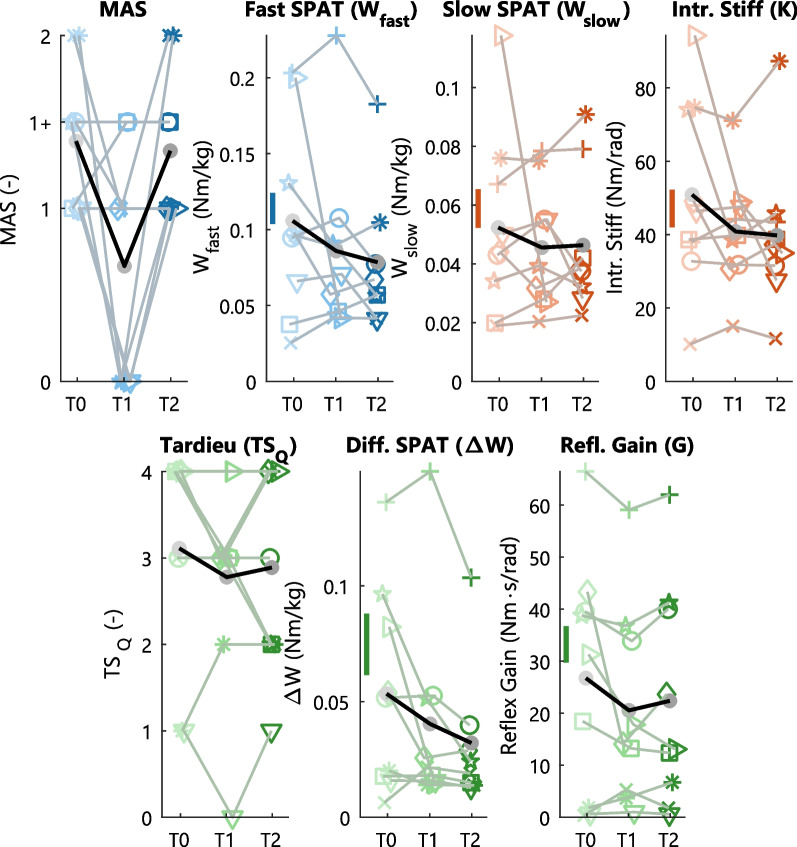
Longitudinal BoNT-A effect on joint resistance contributions for all participants. The quantified joint resistance contributions are shown for each participant *(lines)* at each session *(unique symbol per participant across plots)*: week 0 *(T0, light)*, week 6 *(T1,medium)* and week 12 *(T2, dark)*. The mean values across all participants is shown at each session *(grey dots, bold black lines)*. The BoNT-A effect on overall joint resistance is shown for the MAS (clinical) and $W_{fast}$ (SPAT) *(blue)*. The BoNT-A effect on intrinsic resistance is shown for *(red)*: $W_{slow}$ (SPAT) and intrinsic stiffness (*K*, PC). Finally, the BoNT-A effect on reflexive resistance is shown for *(green)*: the Tardieu Scale (TS_Q_, clinical), $\Delta$Work (SPAT) and reflexive gain (*G*, PC). The best-case minimal detectable difference (MDD) *(vertical line)* is depicted for reference, see Additional file 1: Table S1

The longitudinal evaluation of the BoNT-A effect on joint resistance showed a heterogeneous response across all participants, see Fig. 4 and Table 2.

**Table 2:** Pre- and post-BoNT-A injection outcome measures for the intrinsic and reflexive contributions to ankle joint resistance *(N = 9)*

	T0 (Week 0)	T1 (Week 6)	T2 (Week 12)	Friedman test
MAS (-)	1.5 [1,1.6]	1 [0,1.1]*	1 [1,1.6]	*p* = **0.03**
Fast SPAT $W_{fast}$ (Nm/kg)	0.096 [0.059,0.148]	0.071 [0.045,0.095]	0.062 [0.049,0.091]	*p* = 0.48
Tardieu $TS_Q$ (-)	4 [2.5 4]	3 [2.75,3.25]	3 [2,4]	*p* = 0.52
Diff. SPAT ${\Delta }W$ (Nm/kg)	0.052 [0.017,0.086]	0.022 [0.015,0.052]	0.022 [0.014,0.034]	*p* **= 0.003**
Refl. Gain *G* (Nm$\cdot$s/rad)	31 [1.5,40]	14 [4.9,35]	13 [5.5,40]	*p* = 0.31
Slow SPAT $W_{slow}$ (Nm/kg)	0.044 [0.031,0.069]	0.039 [0.028,0.060]	0.038 [0.030,0.061]	*p* = 0.26
Intr. Stiffness *K* (Nm/rad)	46 [37,74]	40 [32,48]	37 [31,44]	*p* = 0.67

The median [25th, 75th percentile] across participants are reported. Longitudinal differences across all sessions were evaluated using the Friedman test. Significant Friedman tests (${\Delta }W$, MAS) were investigated using a multiple comparisons test adjusted with the Bonferonni correction. Sessions with a significant difference compared with week 0 are indicated (*)

For overall joint resistance, the MAS showed a reduced resistance in 6 of 9 participants at T1 with 5 out of these 6 participants returning to baseline value at T2. The instrumented SPAT overall resistance measure ($W_{fast}$) only showed reduced resistance in 4 participants at T1 with 2 out of these 4 participants returning towards baseline value at T2. On average, both MAS and $W_{fast}$ showed a reduced resistance at T1 with MAS returning close to baseline at T2, whereas $W_{fast}$ showed a further reduction. Only the MAS showed the hypothesized longitudinal BoNT-A effect on group-level ($\chi ^2$[2] = 6.91, *p* = 0.03), with post-hoc comparisons showing a significant reduction between T0 and T1 (t = 2.41, *p* = 0.05). The pROM over which the instrumented assessments were measured changed across sessions in 5 participants. For 2 participants the dorsiflexion pROM was reduced (10$^{\circ }$) at T1, whereas for 3 participants the full pROM shifted (10$^{\circ }$) either towards dorsiflexion (2 participants) or plantarflexion (1 participant). The changes in pROM remained at T2 for 3 participants, whereas 2 participants had a pROM in T2 equal to T0.

For reflexive joint resistance, the Tardieu Scale ($TS_Q$) showed a reduced resistance in 4 participants with 2 out of 4 of these participants returning to baseline value at T2, see Fig. 4 and Table 2. Regarding the instrumented measures a reduction in reflexive resistance at T1 was observed in: 5 participants for ${\Delta }W$, 6 participants for *G*, and 3 participants for both *G* and ${\Delta }W$. Out of these participants with reduced resistance at T1, an increase towards baseline value at T2 was observed in: 1 of 5 participants for ${\Delta }W$, 4 of 6 participants for *G*, and 1 of 3 participants for both *G* and ${\Delta }W$. The participants that did not show a reduction in *G* at T1 had the lowest values for *G* at baseline, see Fig. 4. Combined with the MAS, 4 participants showed reduced resistance at T1 for both MAS and ${\Delta }W$ and 3 participants showed a reduction for both MAS and *G*. On average, all reflexive resistance measures showed a reduction at T1 with both $TS_Q$ and *G* returning towards baseline at T2, whereas ${\Delta }W$ showed a further reduction. A significant longitudinal BoNT-A effect on reflexive resistance was only found for the ${\Delta }W$ ($\chi ^2$[2] = 11.9, *p* = 0.003), although post-hoc comparisons did not find any significant differences between sessions.

For intrinsic joint resistance, a reduced resistance at T1 was observed in: 3 participants for $W_{slow}$, 5 participants for *K*, and 3 participants for both *K* and $W_{slow}$, see Fig. 4 and Table 2. Out of these participants with reduced resistance at T1, an increase towards baseline value at T2 was observed in: 2 of 3 participants for $W_{slow}$, 3 of 5 participants for *K*, and 2 of 3 participants for both *K* and $W_{slow}$. On average, both intrinsic resistance measures showed a reduction at T1 with $W_{slow}$ returning towards baseline at T2 and *K* showing a further reduction. No significant longitudinal BoNT-A effect on intrinsic resistance was found.

**Fig. 5:**
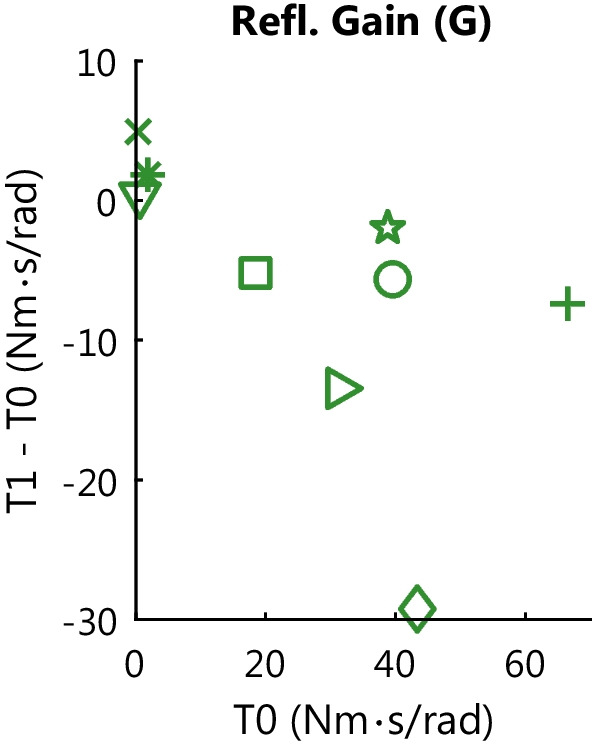
BoNT-A injection effect in relation to baseline value for reflexive joint resistance.The BoNT-A injection effect (difference between week 6 and week 0) (*y-axis*) is shown in relation to the baseline value (*x-axis*) for reflexive gain (*G*, PC). Each symbol represents a single participant, corresponding the symbols used in Fig. 4

The subject heterogeneity likely introduces a confounding effect with the measured BoNT-A injection effect. Participants with high baseline values, i.e. high intrinsic and/or reflexive resistance, show larger responses to the BoNT-A injection than participants with low baseline values, see Fig. 5 with reflexive gain as example outcome measure. Across all measures, moderate to strong correlations between baseline value and measured BoNT-A injection effect were observed, see Table 3. Small baseline values provide little room to observe the hypothesized reduction in joint resistance.

**Table 3:** Spearman’s/Pearson’s correlation coefficient between baseline value (T0) and BoNT-A injection effect (T1–T0) *(N = 8/9)*

Outcome Measure	$\mathbf {\rho }$/$\textbf{r}$
MAS	− 0.51
Fast SPAT $W_{fast}$	− 0.53
Slow SPAT $W_{slow}$	− 0.77
Intr. Stiffness *K*	− 0.81
Tardieu $TS_Q$	− 0.38
Diff. SPAT ${\Delta }W$	− 0.34
Refl. Gain *G*	− 0.57

Spearman’s rank correlation coefficient $\rho$ was used for all correlations involving the ordinal clinical measures, whereas Pearson’s correlation coefficient *r* was used otherwise

### Linear associations and repeatability of joint resistance measures

Excellent ICC values were observed for both the instrumented SPAT ($r=[0.98,0.94,0.97]$) and PC technique ($r=[0.98,0.97,0.99]$) measures, see Table 4. The 95% CIs lower bounds did show relatively high uncertainty for ${\Delta }W$ (0.88), $W_{slow}$ (0.89) and *K* (0.87). The reported ICCs represent a best-case scenario for optimal experimental conditions, as only short 20–60 s breaks were included between repetitions and participants were not taken out of the instrumented setup between repetitions.

**Table 4:** Intraclass correlation coefficient (ICC) and their 95% confidence intervals *(N = 54/78)*

Outcome Measure	ICC
Fast SPAT $W_{fast}$	0.98 [0.96,1.00]
Diff. SPAT ${\Delta }W$	0.94 [0.88,0.98]
Refl. Gain *G*	0.98 [0.97,0.99]
Slow SPAT $W_{slow}$	0.96 [0.89,0.99]
Intr. Stiffness *K*	0.97 [0.87,1.00]
Intr. Damping *B*	0.99 [0.97,1.00]

ICCs for the instrumented assessment based on three repetitions per session for the instrumented SPAT and two repetitions per session for the PC technique. The 95% CIs were constructed using a non-parametric bootstrap procedure

The PC technique model showed a good model fit effectiveness for the overall model and the specific intrinsic and reflexive parameters. Regarding the overall fit (i.e. Step 1–6 of the algorithm), a median variance accounted for (%VAF) of 92.5% [(Q1,Q3) 90.9%, 96.2%] was obtained by the complete model on the measured data, similar to previous PC studies [36]. For the parameterized intrinsic pathway (i.e. Step 4 of the algorithm), a median %VAF of 91.4% [89.0%, 93.1%] was obtained, whereas for the parameterized reflexive pathway (i.e. Step 6 of the algorithm), a median %VAF of 84.4% [77.7%, 87.6%] was obtained.

Most clinical and instrumented assessments quantifying the same resistance contribution showed a positive correlation as expected, see Table 5. For overall resistance, the MAS was not correlated with the SPAT $W_{fast}$ (*r* = 0.05). For the reflexive resistance, the Tardieu Scale showed a moderate positive correlation with the instrumented measures ${\Delta }W$ and *G* (*r* = 0.60/0.57), whereas both instrumented measures showed a strong correlation (*r* = 0.86). For the intrinsic resistance, the SPAT $W_{slow}$ showed a strong correlation with both PC technique outcomes of stiffness *K* (*r* = 0.74) and damping *B* (*r* = 0.71).

**Table 5:** Spearman’s/Pearson’s correlation coefficients and their 95% confidence intervals (*N* = *26–27*)

	MAS	Tardieu $TS_Q$	Refl. Gain *G*	**Intr. Stiffness** *K*	**Intr. Damping** *B*
Fast SPAT $W_{fast}$	0.05 [− 0.38,0.40]	0.24 [− 0.22,0.61]	0.73 [0.48,0.87]	0.46 [0.16,0.78]	0.83 [0.73,0.90]
Diff. SPAT ${\Delta }W$	− 0.01 [− 0.42, 0.41]	0.60 [0.23,0.81]	0.86 [0.71,0.93]	0.17 [− 0.15,0.51]	0.72 [0.43,0.86]
Refl. Gain *G*	− 0.08 [− 0.48, 0.33]	0.57 [0.21,0.80]			
Slow SPAT $W_{slow}$	− 0.03 [− 0.44,0.40]	− 0.09 [− 0.50,0.31]	0.27 [− 0.12,0.59]	0.74 [0.39,0.90]	0.71 [0.41,0.83]
Intr. Stiffness *K*	− 0.20 [− 0.62,0.27]	− 0.07 [− 0.44,0.40]			
Intr. Damping *B*	− 0.04 [− 0.46,0.36]	0.38[− 0.07,0.67]			

Correlations between the clinical measures (MAS, TS_Q_), instrumented SPAT ($W_{fast}$, ${\Delta }W$, $W_{slow}$) and PC technique measures (reflexive gain *G*, intrinsic stiffness *K* and intrinsic damping *B*). Spearman’s rank correlation coefficient $\rho$ was used for all correlations involving the ordinal clinical measures, whereas Pearson’s correlation coefficient *r* was used otherwise. The 95% CIs were constructed using a non-parametric BCa bootstrap procedure

Most clinical and instrumented assessments quantifying a different resistance contribution were not correlated as expected, see Table 5. For the overall resistance, MAS was not correlated with intrinsic/reflexive measures (*r* = [$-$ 0.19, $-$ 0.01]), whereas the SPAT $W_{fast}$ did show strong correlation with the PC reflexive gain *G* and intrinsic damping *B*. For the reflexive resistance, the Tardieu Scale was not or weakly correlated with non-reflexive measures (*r* = [$-$ 0.09, 0.38]). The reflexive gain *G* showed strong correlation with SPAT $W_{fast}$ and the SPAT ${\Delta }W$ showed strong correlation with PC intrinsic damping *G*. For the intrinsic resistance, only PC technique intrinsic damping *B* showed strong correlations as reported above.

## Discussion

This paper studied the intrinsic and reflexive ankle joint resistance within participants treated with BoNT-A injections to reduce spasticity. We hypothesized that both reflexive and overall joint resistance would decrease 6 weeks after BoNT-A injection, while returning close to baseline value after 12 weeks. Three fundamentally different joint resistance assessments were used: (1) clinical tests (MAS, Tardieu Scale); (2) instrumented SPAT measured over the full pROM with elementary processing; and 3) data-driven PC system identification measured over a limited pROM with model-based processing. Individually, the hypothesized BoNT-A effect (reduction at week 6, return to baseline week 12) was observed in the MAS (5 participants), $W_{fast}$ SPAT (2 participants), Tardieu Scale (2 participants), ${\Delta }W$ SPAT (1 participant) and *G* (4 participants). On group-level, our hypothesis was only confirmed for the MAS, a measure of overall joint resistance, which showed a significant reduced resistance at week 6. Regarding validity, all instrumented outcome measures showed a strong correlation when quantifying the same resistance contribution.

### Longitudinal evaluation of BoNT-A injections

On group-level, only the MAS showed the hypothesized effect of reduced joint resistance at week 6 with a return close to baseline at week 12. Our MAS results are in line with larger clinical trials evaluating BoNT-A effects with the MAS [6–8]. The MAS should be interpreted with care as the scale is subjective and a non-blinded rater scored the participants [14, 44]. Contrary to the MAS, all instrumented measures showed a more heterogeneous response and did not capture a significant reduction on group-level 6 weeks after injection. Thus, either there was indeed no significant reduction (true negative) or, as implied by the MAS results and previous clinical trials, we were not able to correctly measure the significant reduction (false negative).

Previous studies using instrumented measures to investigate BoNT-A effects over the full pROM also reported heterogeneity between participants [24–27]. Moreover, a mix of positive/negative results were reported 4–6 weeks after injection for these instrumented assessment studies. The studies executed with a device assessing the wrist (Neuroflexor) and estimating resistance components using a biomechanical wrist model with low complexity did report a reduced reflexive response. The study executed with a device assessing the ankle (MOOG manipulator, similar to our study) and estimating resistance with a neuromechanical ankle model with higher complexity did not report a reduction. Therefore, differences in the reported results may be influenced by participant heterogeneity (such as age, severity of impairment, time since impairment, number of previous BoNT-A injections, BonT-A injection dose), the experimental setup, the assessed joint and the model used for resistance estimation.

The heterogeneous response among the study population complicated group-level evaluation of the BoNT-A effect. For example, the PC technique showed a reflex reduction in 6 out of 9 participants at week 6. The 3 participants without reflex reduction had the lowest reflexive response at baseline. Therefore, these 3 participants had little potential to further reduce the reflexive response and also limited a potential group effect. These 3 participants also had a relatively limited dorsiflexion pROM at baseline and 2 of these 3 participants showed an improved dorsiflexion pROM at week 6. As such, BoNT-A injections may result in better outcomes within people with high reflexive activity and/or clonus than people with only high resistance to passive joint motion. Interpretation of the population heterogeneity was also convoluted by different outcomes for the instrumented measures. A reflex reduction was observed in 5 participants for the SPAT and 6 participants for the PC technique, yet only 3 participants showed a reduction in both outcome measures. As the reflexive response depends on joint angle and pROM, the full and limited pROM used during assessments could potentially explain these differences [36, 45]. Both methods simplified this complex dependency through averaging over the full pROM (SPAT) or assessing a limited pROM (PC). As a result, neither method controlled for variations in the reflexive response due to observed changes in pROM and potential underlying changes in e.g. muscle slack length. Quantitative analysis of the measured individual effects is desired to increase understanding of the heterogeneous response.

Quantitative analysis of individual effects would require a larger participant group and insight into the minimal detectable difference (MDD), which have currently not been reported yet. To illustrate such an analysis, the PC technique showed a reflex reduction larger than a best-case scenario MDD (6.9 Nm s/rad) for 3 of 9 participants at week 6. Only best-case scenario MDDs could be computed as experimental conditions were optimal regarding repeatability. Clinically relevant MDDs would require a test-retest reliability design with longer breaks between repetitions, measurements on separated days and removing participants from the measurement device between repetitions [19, 28, 46]. The best-case results did indeed show that both instrumented SPAT and PC technique had excellent ICC between *r* = [0.94, 0.99], whereas typically reported values are between *r* = [0.85, 0.95] for similar instrumented measures [19, 28, 46–48]. Overall, the BoNT-A effect on the reflexive contributions remains ambiguous.

### Linear associations of joint resistance measures

In absence of a gold standard, the validity of the instrumented measures was shown through linear association between the methodologies [5, 28, 29]. As expected, most measures quantifying the same resistance contribution (e.g. ${\Delta }W$ and *G*) showed moderate to strong correlations. Strong correlations were observed between the instrumented measures, whereas a similar study found moderate similarity between two instrumented measures [29]. However, Andringa et al. [29] compared methodologies using a different experimental setup (Neuroflexor and Wristalyzer) and different data processing approaches (low complexity biomechanical and higher complexity neuromechanical model) [18, 49]. In our study, the results were obtained using the same device, which may explain part of the relatively strong correlations observed.

Only between the MAS and SPAT ($W_{fast}$), both measures of overall joint resistance, no correlation was observed. While both measures compute an overall resistance effect, the characteristics of the applied perturbation differed between the relatively slow velocity of the MAS (20–30 deg/s) and fast velocity of the SPAT, $W_{fast}$ (150 deg/s). Changing perturbation characteristics could affect the relative magnitude of the intrinsic and reflexive contributions within the measured overall response, as both contributions contain velocity- and acceleration-dependent components [10, 50, 51]. Therefore, the lack of association between MAS and fast velocity SPAT could potentially be explained by the different perturbation profiles used. In addition, the MAS, which is a subjective measure, was scored by a non-blinded rater and has questionable reliability, which could all have influenced the observed correlation.

Besides, a general lack of correlation was observed across joint resistance measures quantifying a different resistance contributions, although unexpected correlations were observed between a couple of outcome measures. The reflexive measures of the instrumented SPAT (${\Delta }$W) did show a strong correlation with the intrinsic damping (viscous) contribution of the PC technique (*B*). Note, the reflexive instrumented SPAT measure was computed as the difference in work between a fast and slow passive movement. Thus, ${\Delta }$W was considered fully velocity-dependent, which can be attributed to either a reflexive or viscous intrinsic contribution [10]. This could explain the observed commonality with intrinsic damping of the PC technique. The commonality of the reflexive SPAT measures with an intrinsic outcome measure illustrated that the separation of joint resistance contributions could be improved. On the one hand, additional information from an extended experimental dataset might improve the ability to disentangle joint resistance. On the other hand, detailed model-based processing, such as neuromechanical models or data-driven processing, could improve the ability to disentangle joint resistance [10, 24–27]. Andringa et al. [29] did show that despite the use of these type of neuromechanical models, weak correlations between reflexive and intrinsic contribution may remain. Overall, at group-level the quantified intrinsic and reflexive resistance outcome measures matched well, supporting the validity towards clinical application.

### Study limitations and clinical application

First, the clinical evaluations in this study were all performed by a non-blinded, trained physiotherapist. Therefore, knowledge of the hypotheses of this study combined with information about the specific session (week 0, 6 or 12) could have biased the MAS and Tardieu Scale scores. Second, spasticity is a complex symptom, which can manifest itself differently within a the passive experimental environment compared with an active or functional environment [4]. Therefore, BoNT-A effects as experienced in daily life and functional tasks may not necessarily be captured in the clinical and instrumented assessments used. In addition, the full complexity of spasticity is difficult to capture within the limited number of participants included in the study. Third, a low reflexive resistance magnitude at baseline before BoNT-A injection was observed in 3 participants, which limited their potential to show a reflex reduction. Scientifically, future studies evaluating longitudinal BoNT-A effects could avoid this limitation by determining a threshold magnitude, e.g. based on MDD, for inclusion of participants in the data analysis. Clinically, these 3 participants illustrate the relevance of adding instrumented measures to enable differentiation between patients with similar MAS values in support of clinical decision making. Fourth, the instrumented evaluations were limited due to natural variations in the pROM shown by multiple participants across sessions. Small variations in pROM were exacerbated in our protocol, because the adjustable hardware endstops restricting manipulator movement for safety could only be adjusted per $10^{\circ }$. For both instrumented measures, variability in the pROM likely translated to additional variability in outcome measures across sessions, as joint resistance depends on joint angle and pROM [36, 45]. Due to simplification in both instrumented measures, the added variability of the pROM could not be controlled for, which reduced the ability to detect BoNT-A effects.

Despite these limitations and heterogeneous results, clinical studies of instrumented measures distinguishing the relative contributions to joint hyper-resistance remain important. First, our results again confirm that the MAS, on which many clinical evaluations of BoNT-A effects are based, does not correlate well with instrumented measures specifically aimed at quantifying the reflexive joint resistance or spasticity. Second, further research into the diagnostic properties of the instrumented measures is of interest to potentially support clinical decision making. For example, previous studies showed that the PC technique could discriminate spastic participants from controls and paretic from non-paretic joints [30, 32]. Towards clinical application, additional investigation into diagnostic properties like the reliability (MDD) and normative data are desired to enable clinical decision making based on the quantified joint resistance contributions. Moreover, investigating the relation between instrumented measures and functional or goal-oriented outcomes, like motor recovery level or goal attainment scale (GAS) [52], is of interest. This relation is important: given the lack of a gold standard to evaluate the instrumented measures against; to examine the role of increased intrinsic and reflexive joint resistance on reduced functionality; and to provide extended clinical context on the indication and evaluation of BoNT-A treatment.

## Conclusions

Our group-level hypothesis of a reduced joint resistance 6 weeks after injection with a return close to baseline at week 12 was only observed in the MAS (overall joint resistance). This observed reduction could not be attributed to an unambiguous group-level reduction of the reflexive or intrinsic resistance as no instrumented measures confirmed the hypothesis. Several individuals did show the hypothesized BoNT-A effect in the reflexive or intrinsic contributions. A moderate to strong correlation between all reflexive measures and a strong correlation between the intrinsic measures supported the validity of the used instrumented measures. Ultimately, objective and reliable joint resistance quantification would improve clinical decision making in prescription of BoNT-A and unravel the effect of BoNT-A injections on spasticity.

## Supplementary Information

**Additional file 1.** Best-case minimal detectable difference for the intrinsic and reflexive joint resistance measures.

## Data Availability

The code and data underlying this publication are available via 4TU.ResearchData: 10.4121/c.5986267.

## Abbreviations

AIS: American spinal injury association (ASIA) impairment scale
BoNT-A: Botulinum neurotoxin-A
CI: Confidence interval
GAS: Goal attainment scale
GM: Gastrocnemius medialis
GL: Gastrocnemius lateralis
IC: Intraclass correlation coefficient
IRF: Impulse response function
MAS: Modified Ashworth scale
MDD: Minimal detectable difference
PC: Parallel-cascade
pROM: Passive range of motion
SCI: Spinal cord injury
SOL: Soleus
SPAT: Spasticity test
TP: Tibialis posterior
VAF: Variance accounted for
