# Supplementary material for: Examining the role of intrinsic and reflexive contributions to ankle joint hyper-resistance treated with botulinum toxin-A
Source: J Neuroeng Rehabil. 2023 Feb 7;20:19. doi: 10.1186/s12984-023-01141-8 (PMC9906865; doi:10.1186/s12984-023-01141-8)
Supplement: Supplementary file 1 — Additional file 1. Best-case minimal detectable difference for the intrinsic and reflexive joint resistance measures. [file 12984_2023_1141_MOESM1_ESM.pdf]

Additional file 1

Table S1 Best-case minimal detectable difference (MDD) (N = 54/78)

Outcome Measure	MDD
Diff. SPAT ΔW (Nm/kg)	0.026
Slow SPAT W_{slow} (Nm/kg)	0.013
Fast SPAT W_{fast} (Nm/kg)	0.021
Refl. Gain G (Nm s/rad)	6.9
Intr. Stiffness K (Nm/rad)	10
Intr. Damping B (Nm s/rad)	0.094

MDDs for the instrumented assessment outcomes based on the ICC values in Table 4 [52]. The MDD present a best-case scenario as the repeatability was tested under most optimal circumstances.
